# Subconjunctival administration of low-dose murine allogeneic mesenchymal stromal cells promotes corneal allograft survival in mice

**DOI:** 10.1186/s13287-021-02293-x

**Published:** 2021-04-06

**Authors:** Oliver Treacy, Kevin Lynch, Nick Murphy, Xizhe Chen, Ellen Donohoe, Aoife Canning, Paul Lohan, Georgina Shaw, Gerry Fahy, Aideen E. Ryan, Thomas Ritter

**Affiliations:** 1 College of Medicine, Nursing and Health Sciences, Biomedical Sciences, Regenerative Medicine Institute, National University of Ireland Galway, Galway, Ireland; 2 Discipline of Pharmacology and Therapeutics, College of Medicine, Nursing and Health Sciences, National University of Ireland Galway, Galway, Ireland; 3 Department of Ophthalmology, University Hospital Galway, National University of Ireland Galway, Galway, Ireland; 4 CURAM Centre for Research in Medical Devices, National University of Ireland, Galway, Ireland

**Keywords:** Corneal transplantation, Mesenchymal stromal cells, Allogeneic, Subconjunctival, Immunomodulation, Macrophage, Graft survival, Mouse

## Abstract

**Background:**

Systemic administration of mesenchymal stromal cells (MSCs) has been efficacious in many inflammatory disease settings; however, little data are available on the potential immunomodulatory effects following local MSC administration in the context of corneal transplantation. The purpose of this study was to assess the potential of subconjunctival injection of MSCs to promote corneal allograft survival.

**Methods:**

MSCs were isolated from female C57BL/6 (H-2^k^) or Balb/c (H-2^d^) mice and extensively characterized. An allogeneic mouse corneal transplant model was used with Balb/c mice as recipients of C57BL/6 grafts. A dose-finding study starting with 5 × 10^5^ MSCs injected subconjunctivally at day − 7 was tested first followed by a more clinically translatable low-dose single or dual injection strategy on day − 1 and day + 1 before/after transplantation. Graft transparency served as the primary indicator of transplant rejection while neovascularization was also recorded. Lymphocytes (from draining lymph nodes) and splenocytes were isolated from treatment groups on day 2 post-transplantation and characterized by flow cytometry and qRT-PCR.

**Results:**

Both high- and low-dose injection of allogeneic MSCs on day − 7 led to 100% graft survival over the observation period. Moreover, low-dose dual subconjunctival injection of 5 × 10^4^ allogeneic MSCs on day − 1 or day + 1 led to 100% allograft survival in transplant recipients (*n* = 7). We also demonstrate that single administration of allogeneic MSCs on either day − 1 or day + 1 promotes rejection-free graft survival in 100% (*n* = 8) and 86% (*n* = 7) of transplanted mice, respectively. Early time point ex vivo analysis suggests modulation of innate immune responses towards anti-inflammatory, pro-repair responses by local MSC administration.

**Conclusion:**

This work demonstrates that low-dose subconjunctival injection of allogeneic MSCs successfully promotes corneal allograft survival and may contribute to refining future MSC immunotherapies for prevention of corneal allograft rejection.

## Introduction

Corneal transplantation is the last option for patients suffering from serious ocular disease or injury [1–3]. Topical corticosteroids with or without adjuvant immunosuppressant therapy remains the gold standard treatment for preventing corneal allograft rejection; however, patients are still highly susceptible to immune-mediated rejection. Therefore, novel therapies are urgently needed to improve the prognosis of corneal transplantation.

Mesenchymal stromal cells’ (MSCs) therapeutic potential is associated with their ability to effectively modulate host repair responses and inflammation and their safety profile following administration to patients [4, 5]. We and others have shown that systemic administration of MSCs prolongs corneal allograft survival and promotes ocular surface regeneration [6–10]. Although the mechanisms of MSC-induced immunomodulation in vivo are not completely understood, data suggest that induction/expansion of regulatory T cells (Tregs) or myeloid-derived suppressor cells in the lung [7, 11] play an important role. Most pre-clinical and clinical studies administer MSCs systemically; however, this often requires high doses of cell numbers to achieve a therapeutic effect, which may lead to adverse side effects [12, 13]. In contrast, local application of MSCs may have the potential to exert local immunomodulatory effects, which may allow for the application of reduced cell numbers. Subconjunctival injection of MSCs (albeit large numbers of cells) seems to be beneficial in cornea injury models [14, 15] and in a rat corneal allotransplant model [16]. Here, we show that, following subconjunctival administration, allogeneic donor-derived but not syngeneic MSCs promote corneal allograft survival in a dose-dependent manner. Our data indicate that a single bolus of locally administered MSCs is sufficient to significantly attenuate corneal allograft rejection. Moreover, early time point ex vivo analysis suggests modulation of innate immune responses by local MSC administration.

## Materials and methods

### Mouse corneal transplantation

A fully allogeneic major histocompatibility complex (MHC) class I/II disparate cornea transplant model was used for these studies as described previously [10]. See Additional file 1 for details.

### Generation and characterization of mouse MSCs

Isolation of mouse MSCs was performed as described [10], and MSC preparations were extensively characterized for the expression or absence of specific cell surface markers by flow cytometry and for their differentiation capacity (Supplemental Figure 1 and 2) [7, 10, 17, 18].

### RAW264.7 macrophage/MSC co-culture assay

See Additional file 1 for details.

### Subconjunctival administration of MSCs

MSCs were collected, washed three times with DPBS (Thermo-Fisher Scientific, Dublin, Ireland), and filtered through a 40-μm filter (Thermo-Fisher Scientific) before administration. Mice were briefly anesthetized using isoflurane and subconjunctivally administered 5 × 10^4^ (low-dose) or 5 × 10^5^ (high-dose) MSCs in 20 μl or 50 μl of DPBS, respectively, using a 30-G needle.

### Generation of single-cell suspensions from lymph nodes and spleens and flow cytometry

Single-cell suspensions of draining lymph nodes (dLNs) and spleens were prepared as detailed in [10]. See Additional file 1 for details.

### RNA isolation and RT-PCR

See Additional file 1 for details.

### Statistical analysis

See Additional file 1 for details.

## Results

### Subconjunctival injection of allogeneic MSCs promotes corneal allograft survival in Balb/c mice

MSCs were isolated from C57BL/6 and Balb/c mice as previously described and extensively characterized [10, 17]. MSCs were shown to conform to ISCT criteria (Supplemental Figure 1 and 2). Data relating to characterization and differentiation of Balb/c MSCs can be found at [10].

**Fig. 1:**
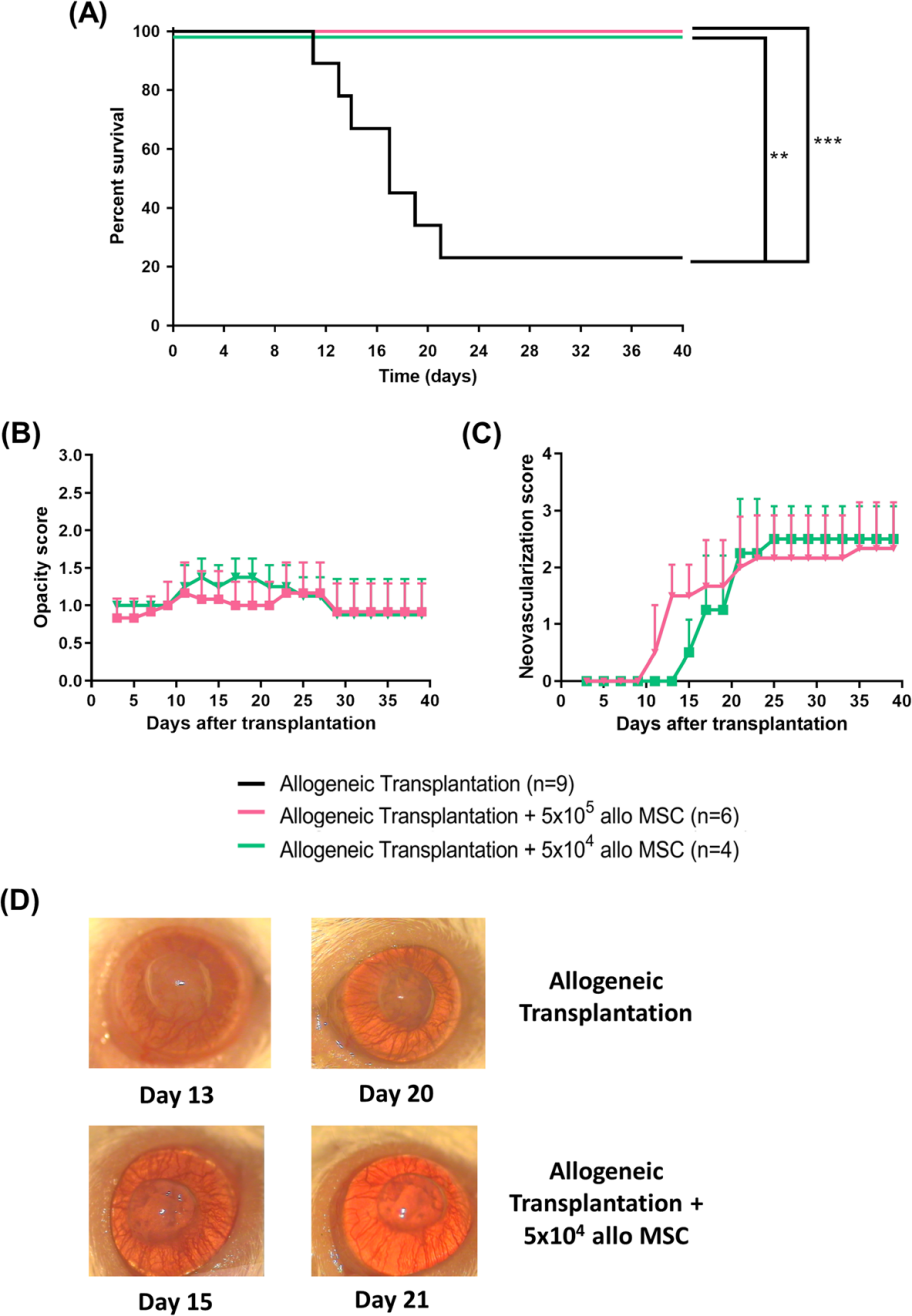
Subconjunctival administration of allogeneic MSCs prolongs corneal allograft survival. Female Balb/c mice served as recipients for female C57BL/6 donor corneas. Different doses of allogeneic (donor-derived) MSCs were injected subconjunctivally 7 days before transplantation (D−7). Mice were observed every 2/3 days. **a** Kaplan-Meier survival curve analysis of allogeneic transplant controls (black line) (*n* = 9), corneal allograft + 5 × 10^5^ MSCs (pink line) (*n* = 6), and corneal allograft + 5 × 10^4^ MSCs (green line) (*n* = 4) (Log-rank (Mantel-Cox) test, **p ≤ 0.01, ***p ≤ 0.001). **b** Opacity and **c** neovascularization scores up to POD 40. **d** Representative light microscopy images of corneal allografts taken at two post-transplantation time points from two separate mice either treated with low-dose allo-MSCs or untreated. *n* = 4–6 with numbers per treatment group the same as in **a**. Error bars show mean + SD

Next, we investigated if local administration of MSCs could promote mouse corneal allograft survival by applying two different injection strategies with either one or two MSC injections. First, recipient Balb/c mice received either no injection or a single bolus injection of 5 × 10^5^ (high-dose) or 5 × 10^4^ (low-dose) allogeneic C57BL/6 MSCs in the subconjunctival space followed by allogeneic corneal transplantation 7 days later. Transplant survival was monitored over a period of 40 days by microscopy and graft opacity as the main indicator of cellular infiltration and endothelial dysfunction was recorded. The majority of allogeneic control transplanted mice rejected their corneal transplant before the end of the observation period (mean survival time (MST), 21.3 days ±11.0 SD). In contrast, animals that received a bolus of 5 × 10^5^ MSCs had a 100% survival rate (Fig. 1a). Interestingly, low-dose (5 × 10^4^) was equally as efficacious as high-dose treatment (Fig. 1a). Earlier onset of neovascularization was observed in the high-dose allo-MSC group (Fig. 1c), but no significant difference in opacity was seen between mice receiving either low- or high-dose allo-MSCs (Fig. 1b). Representative images of low-dose MSC-treated and allogeneic transplant control mice are shown in Fig. 1d.

**Fig. 2:**
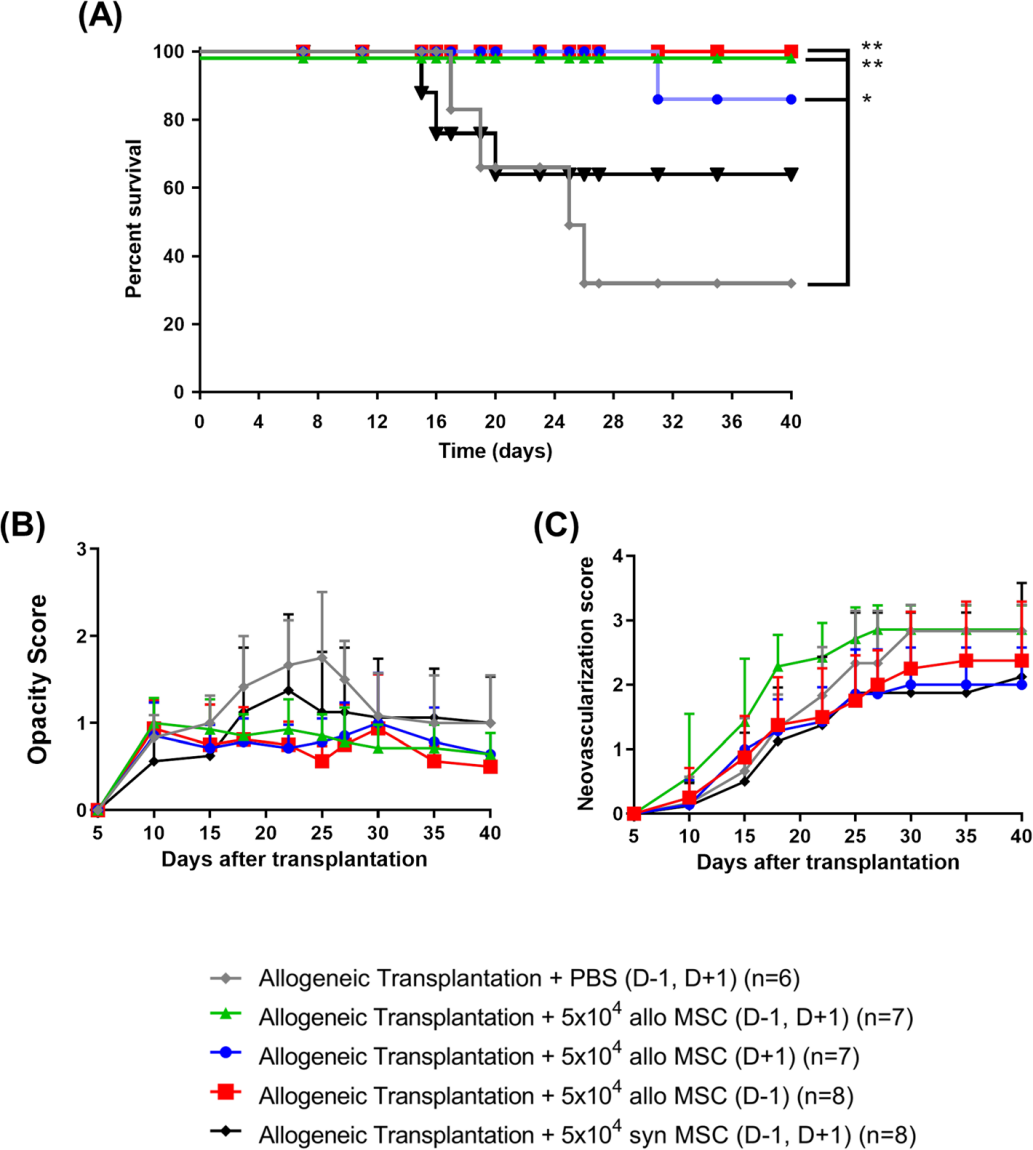
Subconjunctival administration of low-dose allogeneic MSCs prolongs corneal allograft survival with pre-transplant injection the crucial factor. Female Balb/c mice served as recipients for female C57BL/6 donor corneas. Allogeneic MSCs were administered subconjunctivally using three different treatment strategies. Mice received one injection of 5 × 10^4^ MSCs either the day before transplantation (D−1) or the day after transplantation (D+1) or two separate doses at D−1 and D+1. Control mice received PBS alone at D−1 and D+1. Mice were observed every 2/3 days. **a** Kaplan-Meier survival curve analysis of corneal allograft + PBS (D−1, D+1)-treated control mice (gray line) (*n* = 6), corneal allograft + 5 × 10^4^ allogeneic MSCs (D−1, D+1) (green line) (*n* = 7), corneal allograft + 5 × 10^4^ allogeneic MSCs (D+1) (blue line) (*n* = 7), corneal allograft + 5 × 10^4^ allogeneic MSCs (D−1) (red line) (*n* = 8), and corneal allograft + 5 × 10^4^ syngeneic MSCs (D−1, D+1) (black line) (*n* = 8) (Log-rank (Mantel-Cox) test, *p ≤ 0.05, **p ≤ 0.01). **b** Opacity and **c** neovascularization scores up to POD 40. *n* = 6–8 with numbers per treatment group the same as in **a**. Error bars show mean + SD

Following confirmation that local administration of low-dose MSCs was just as efficacious as high-dose treatment, we modified our injection strategy towards a more clinically feasible injection protocol. We investigated if subconjunctival MSC injection on the day before and/or after the day of transplantation could promote corneal allograft survival. We found that double injection of low-dose MSCs on day − 1 and day + 1 led to 100% corneal allograft survival (Fig. 2a). Interestingly, a single injection strategy either on day − 1 or day + 1 also resulted in significant prolongation of allograft survival (Fig. 2a). While not reaching statistical significance, there was a clear trend towards reduced corneal opacity in all MSC treatment groups versus PBS-treated control mice (PBS v allo-MSC (D−1, D+1): *p* = 0.0717; PBS v allo-MSC (D−1): *p* = 0.0699; PBS v allo-MSC (D+1): *p* = 0.0641) (Fig. 2b). Neovascularization levels were comparable between all groups (Fig. 2c). Interestingly, dual injection of syngeneic, recipient-derived MSCs had no significant impact on corneal allograft survival (Fig. 2a).

### Early time point analysis of draining lymph nodes following subconjunctival injection of MSCs indicates innate immune cell modulation

**Fig. 3:**
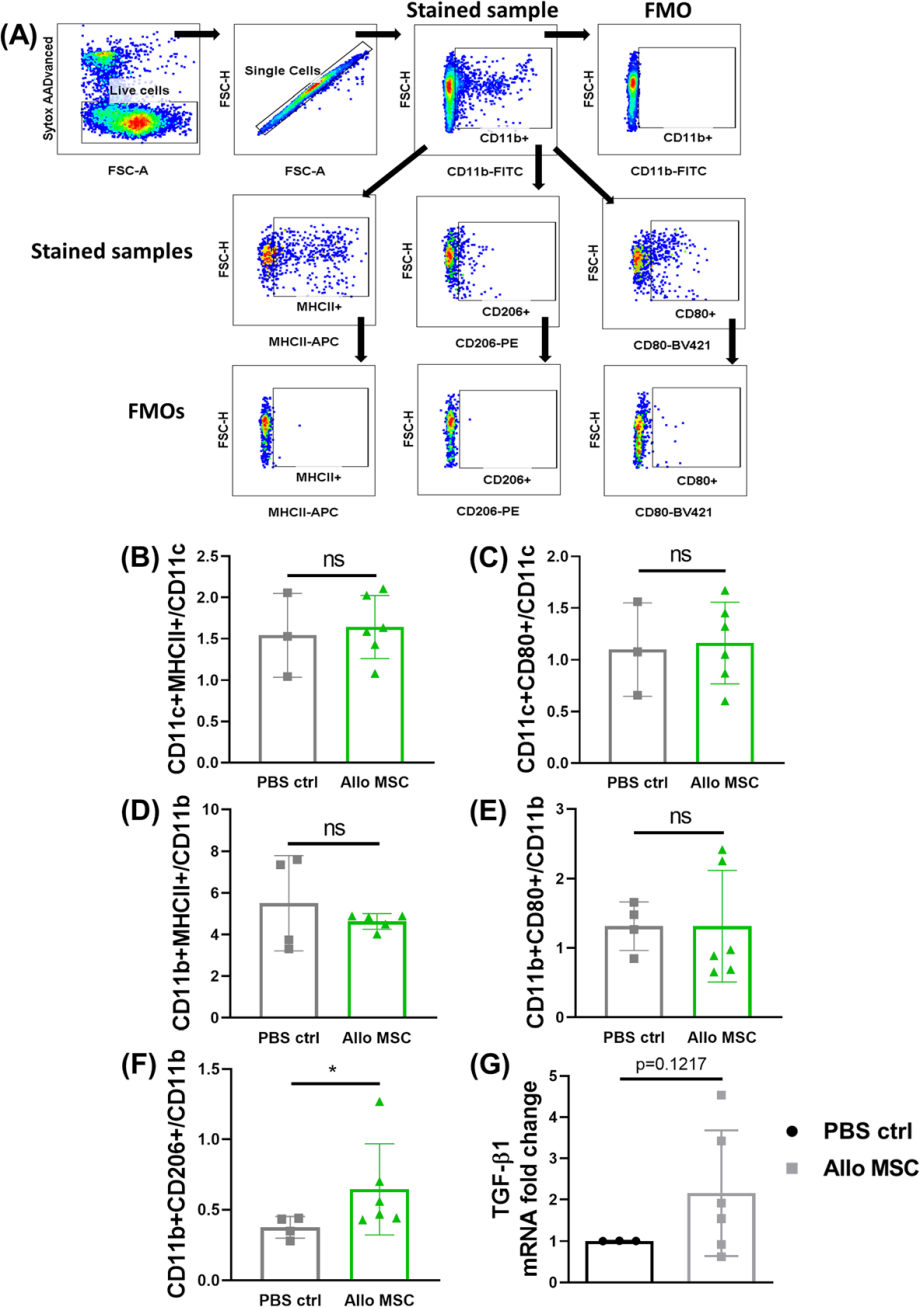
Dual administration of low-dose allogeneic MSCs induces a higher proportion of anti-inflammatory mononuclear phagocytes in the draining lymph nodes. Draining lymph nodes (dLNs) were harvested 2 days post-transplantation (D+2) from corneal allograft recipient mice receiving either two injections of PBS or low-dose allogeneic MSCs the day before transplantation (D−1) and the day after transplantation (D+1). **a** Flow cytometry gating strategy used to select activated dendritic cells (DCs) (CD11c+MHCII+, CD11c+CD80+) or mononuclear phagocytes (MPh) with either a pro-inflammatory (CD11b+MHCII+, CD11b+CD80+) or an anti-inflammatory (CD11b+CD206+) phenotype. **b** Proportion of MHCII+ DCs expressed as a percentage of the parent (CD11c+) population. **c** Proportion of CD80+ DCs expressed as a percentage of the parent (CD11c+) population. **d** Proportion of MHCII+ MPh expressed as a percentage of the parent (CD11b+) population. **e** Proportion of CD80+ MPh expressed as a percentage of the parent (CD11b+) population. **f** Proportion of CD206+ MPh expressed as a percentage of the parent (CD11b+) population. **g** Analysis of mRNA expression (normalized to the housekeeping gene GAPDH and shown as fold-change relative to the PBS-treated allogeneic control group) in the dLNs of TGF-β1 at D+2 from PBS-treated allogeneic controls and low-dose allogeneic MSC-treated corneal allograft recipients. Error bars: mean ± SD. *p < 0.05 (each individual dot represents a separate animal, *n* = 3–6). D’Agostino and Pearson omnibus normality test and Shapiro-Wilk normality test used to determine the distribution of data. ROUT testing was used to identify outliers. Non-parametric unpaired two-tailed Student’s *t* tests used for data that was not normally distributed

**Fig. 4:**
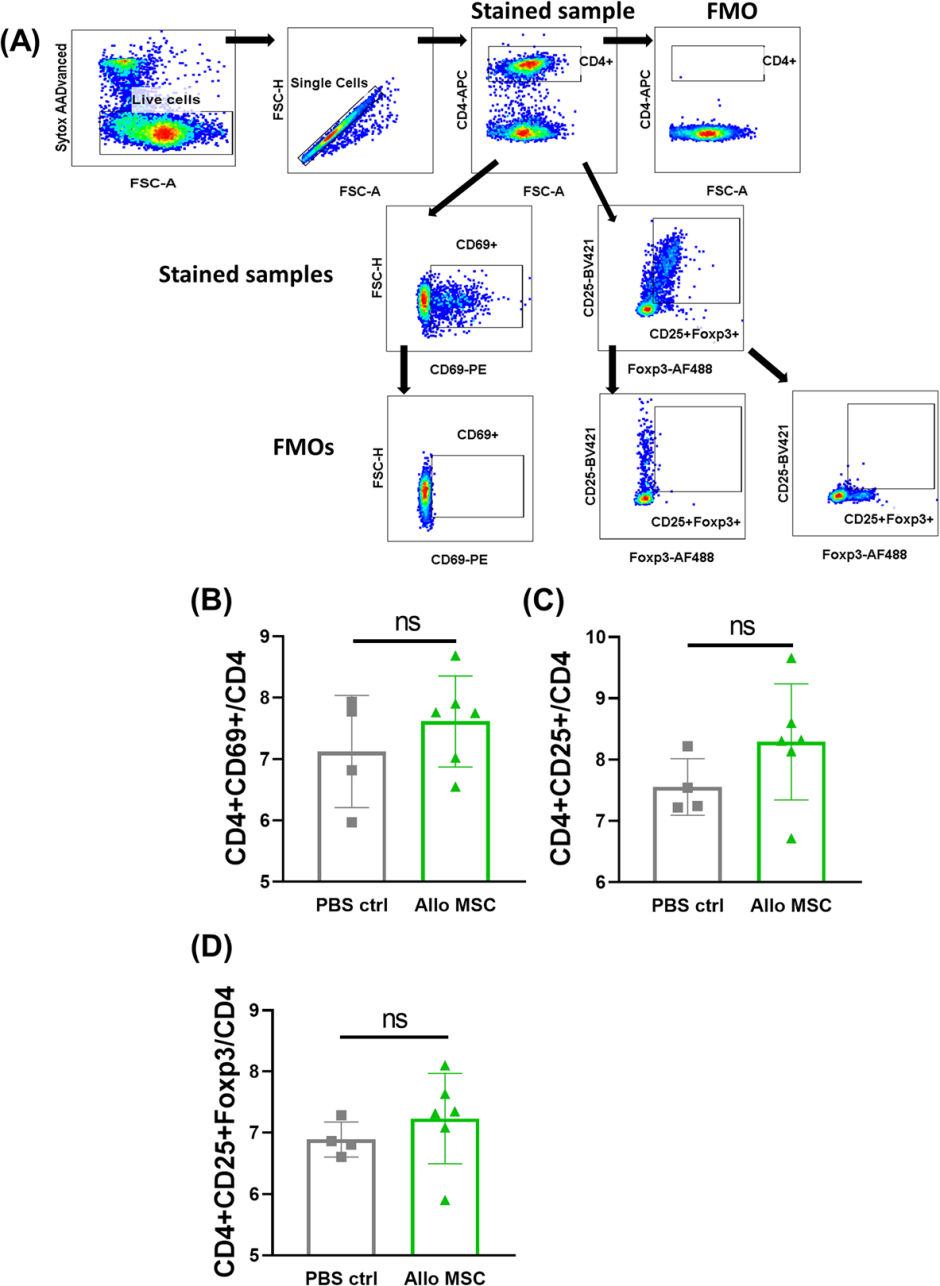
Dual administration of low-dose allogeneic MSCs does not significantly alter the frequency of activated CD4+ T cells or regulatory T cells in the draining lymph nodes. Draining lymph nodes (dLNs) were harvested at D+2 from corneal allograft recipient mice receiving either two injections of PBS or low-dose allogeneic MSCs (D−1 and D+1). **a** Flow cytometry gating strategy used to select activated CD4+ T cells (CD4+CD25+ or CD4+CD69+) or regulatory T cells (Tregs) (CD4+CD25+Foxp3+). **b** Proportion of CD69+ T cells expressed as a percentage of the parent (CD4+) population. **c** Proportion of CD25+ T cells expressed as a percentage of the parent (CD4+) population. **d** Proportion of CD25+Foxp3+ Tregs expressed as a percentage of the parent (CD4+) population. Error bars: mean ± SD (each individual dot represents a separate animal, *n* = 4–6). D’Agostino and Pearson omnibus normality test and Shapiro-Wilk normality test used to determine distribution of data. ROUT testing was used to identify outliers. Non-parametric unpaired two-tailed Student’s *t* tests used for data that was not normally distributed

As it is well known that the draining lymph nodes (dLNs) play an important role in corneal allograft survival and rejection, we investigated the immunological profile of lymph node cells following dual MSC injection on day − 1/+ 1. MSC-treated transplanted animals were sacrificed 2 days post-transplantation, and LN cells were isolated and profiled by flow cytometry and qRT-PCR. Although we found no significant differences in MHC class II and CD80 expression on CD11b+ and CD11c+ cells (Fig. 3b–e) (see Fig. 3a for gating strategy) between the two groups, we found that the proportion of CD206-expressing CD11b+ cells (indicative of non-classical, anti-inflammatory M2-like macrophages) was significantly increased in the dLNs of mice treated with two doses of allo-MSCs (Fig. 3f). qRT-PCR analysis of dLNs also showed a trend towards an increase in TGF-β mRNA expression (though not statistically significant) in allo-MSC-treated animals (Fig. 3g). Analysis of the same immune cell subsets in the spleen revealed no significant differences between allo-MSC and PBS control treated transplanted mice (Supplemental Figure 3A-E). This would suggest that the observed increased proportion of CD11b+CD206+ cells is a LN-specific finding. To further validate that MSCs (specifically allo-MSCs) can induce a M2-like anti-inflammatory macrophage phenotype, we co-cultured RAW264.7 macrophages with either syngeneic (Balb/c) or allogeneic MSCs (C57BL/6) for 72 h (see Supplemental Figure 4A for experimental outline). Allo-MSCs were capable of both polarizing M0 macrophages (Supplemental Figure 4B) and skewing M1-polarized macrophages (Supplemental Figure 4C) to a M2-like phenotype, as measured by fold expression of the M2 marker arginase-1 (Arg-1). There was also a clear increase in Arg-1 expression by M2-polarized macrophages following co-culture with allo-MSCs (Supplemental Figure 4D). Confirmation that Arg-1 expression was coming from the macrophages and not the MSCs themselves was obtained by assessing Arg-1 expression by the MSCs alone. As shown in Supplemental Figure 4E, neither MSC population expressed Arg-1 mRNA at a physiologically relevant level. While not reaching statistical significance, analysis of early CD4+ T cell responses showed trends towards increased levels of both CD69+ (early marker of activation) (Fig. 4b) and CD25+ (Fig. 4c) expressing T cells (see Fig. 4a for gating strategy). This is perhaps reflective of the early time point post-transplantation at which the analysis was performed. CD4+CD25+Foxp3+ Treg levels were comparable between both groups (Fig. 4d). In summary, these data show that local administration of allo-MSCs promotes corneal allograft survival and modulates innate immune cell populations in the dLNs towards a more anti-inflammatory/regulatory phenotype early after MSC injection.

## Discussion

The field of MSC therapies has grown exponentially over the last decade with MSCs being applied in many disease settings but with mixed beneficial results [4, 13]. Many important issues for the success of clinical applications of MSCs such as timing of cell administration, dose, and origin of MSCs are still not solved satisfactorily, therefore warranting further in vitro and pre-clinical investigations. Here we show that local, subconjunctival injection of allogeneic MSCs is as efficient as intravenous injection in modulating corneal allograft survival [7, 19] but at very low doses (5 × 10^4^ MSCs). This is significant as this novel low-dose injection protocol may also reduce the risk of potential side effects of allogeneic cell administration in patients. Moreover, it will allow a much larger number of patients to be treated per donor bone marrow, thereby contributing to reducing the costs of cell manufacturing for clinical trials. Interestingly, both tested subconjunctival injection strategies using different time points (day − 7 or day − 1/+ 1) resulted in equally efficient prevention of corneal transplant rejection, indicating that the time point of cell administration is less important in this disease setting. Of note, double injection of syngeneic/autologous (recipient-derived) MSCs did not lead to significant prolongation of allograft survival, a phenomenon which we have previously described [7, 10]. Immunomodulatory effects following intravenous infusion of MSCs can be regulated through the myeloid cell-mediated induction of Foxp3^+^ regulatory T cells in the lung, a phenomenon which was first described by Ko et al. [11] and confirmed by us and others [9, 10, 20, 21]. Interestingly, the injection of 5 × 10^4^ MSCs in the subconjunctival space is unlikely to result in these MSCs migrating to the lung to exert their therapeutic effect; therefore, other mechanisms of MSC immunomodulation are likely at play. It is likely that MSCs not only have the capacity to modulate monocytes/macrophages in the lung but also in other immunorelevant compartments such as in the eye, the conjunctiva-associated lymphoid tissues (CALT), or in the draining lymph nodes. Indeed, we found that subconjunctival injection of MSCs leads to detectable changes in the expression profile of innate immune cells in the draining lymph nodes as early as 2 days after transplantation, resulting in a significant upregulation of CD206 expression on mononuclear phagocytes. This suggests that 1–3 days after MSC administration, a skewing of the anti-graft immune response towards a more anti-inflammatory or wound healing phenotype has occurred.

In conclusion, we have shown that the subconjunctival administration of MSCs has the potential to significantly prolong corneal allograft survival using a single injection strategy and a much reduced MSC dose compared to systemic administration. This novel injection protocol could lead to the development of novel therapeutic treatment regimens for patients who suffer from adverse immune reactions towards an allogeneic corneal transplant and with much reduced cell numbers enhancing safety and efficacy of cellular therapies.

## Supplementary Information

**Additional file 1: Table S1.** Antibodies used for flow cytometry.

**Additional file 2: Supplemental Figure 1**. Surface profile characterization of C57BL/6 MSCs.

**Additional file 3: Supplemental Figure 2**. Osteogenic and adipogenic differentiation of C57BL/6 MSCs.

**Additional file 4: Supplemental Figure 3.** Dual administration of low-dose allogeneic MSCs does not significantly alter the frequency of mononuclear phagocytes or activated dendritic cells in the spleen.

**Additional file 5: Supplemental Figure 4.** C57BL/6 MSCs polarize M0 and skew M1-like macrophages towards an-anti-inflammatory, M2-like phenotype after co-culture.

## Data Availability

The datasets used and/or analyzed during the current study are available from the corresponding author on reasonable request.

## Abbreviations

MSCs: Mesenchymal stromal cells
Tregs: Regulatory T cells
MHC: Major histocompatibility complex
DPBS: Dulbecco’s phosphate-buffered saline
dLNs: Draining lymph nodes
ISCT: International Society for Cell Therapy
MST: Mean survival time
Arg-1: Arginase-1
CALT: Conjunctiva-associated lymphoid tissues
POD: Post-operation day
MPh: Mononuclear phagocytes
DCs: Dendritic cells
