# Supplementary material for: Subconjunctival administration of low-dose murine allogeneic mesenchymal stromal cells promotes corneal allograft survival in mice
Source: Stem Cell Res Ther. 2021 Apr 6;12:227. doi: 10.1186/s13287-021-02293-x (PMC8025388; doi:10.1186/s13287-021-02293-x)
Supplement: Supplementary file 1 — Additional file 1: Table S1. Antibodies used for flow cytometry. [file 13287_2021_2293_MOESM1_ESM.docx]

**Supplemental File**

**Methods**

**Mouse corneal transplantation**

All procedures performed on mice were approved by the Animals Care Research Ethics Committee of the National University of Ireland, Galway (NUIG) and conducted under individual and project authorisation licenses from the Health Products Regulatory Authority (HPRA) of Ireland. All animals were housed and cared for under Standard Operating Procedures of the Animal Facility at the Biomedical Sciences Biological Resource Unit, NUIG. Female C57BL/6 (H-2^b^) mice served as corneal graft donors and female Balb/c (H-2^d^) mice served as recipients. All animals were aged between 8-14 weeks old and obtained from Envigo Laboratories (Oxon, UK) and housed with food and water ad lib. Orthotopic corneal transplantation was performed as follows. Briefly, animals were anaesthetised by placing in an anaesthesia box connected to an isoflurane vaporizer and pre-filled with a mixture of oxygen and isoflurane (5% anaesthetic in 2 L/min medical oxygen (BOC Gases, Galway, Ireland)). For surgical anaesthesia, a mixture of ketamine (90mg/kg) and xylazine (7.5mg/kg) was injected intraperitoneally. Deep anaesthesia was achieved when limb withdrawal and eye reflexes were abolished. Depth of anaesthesia was monitored by the breathing pattern of the animals. 1% tetracaine (Chauvin Pharmaceuticals, Kingston upon Thames, UK) was administered as a local anaesthetic and 1% atropine sulphate, 1% tropicamide and 2.5% phenylephrine hydrochloride (all Chauvin Pharmaceuticals) were administered for pupil dilation. A 1.5mm graft bed was prepared and a 2mm donor graft was sutured in place with a continuous looped 11-0 Ethilon suture (Ethicon, Livingston, Scotland). Antibiotic ointment containing chloramphenicol was applied to the graft. Irrigation of the corneal tissue was achieved by application of balanced salt solution (BSS) (Alcon, Hemel Hempstead, UK). Eyelids were sutured closed to prevent the animals from scratching the graft and were removed two days after surgery. Corneal sutures were removed seven days after surgery. Graft opacity as the primary indicator of rejection was scored every 2-3 days using a Leica operating microscope at 25x magnification and graded on a scale of 0-3, with 0 being a completely transparent cornea and 3 corresponding to complete corneal opacity, anterior chamber not visible. A graft was considered to be rejected when an opacity score of 2 was recorded on two consecutive days or one score of 2.5 or above. Animals with surgical complications were excluded. Neovascularization was also assessed and quantified by evaluating the number of quaternary segments of donor corneas in which vessels were present (10).

**RAW264.7 macrophage/MSC co-culture assay**

Balb/c-derived RAW264.7 macrophages were seeded at a concentration of 1x10^5^ cells per well in 6 well plates in macrophage complete medium (consisting of DMEM (Bio-Sciences, Dublin, Ireland), 10% heat-inactivated FBS, and 1% penicillin (10U/ml)/streptomycin (100 mg/mL) (both Sigma-Aldrich, Wicklow, Ireland) and allowed to adhere for 6h. For M1-polarization, media was aspirated and replaced with macrophage complete medium containing 100U/ml of recombinant murine IFN-γ (Cat. no. 315-05, Peprotech, London, UK) for 24h followed by 10ng/ml of lipopolysaccharide (LPS) (Cat. no. L2630, Sigma-Aldrich) stimulation for 4h. For M2-polarization, media was aspirated and replaced with macrophage complete medium containing 10ng/ml of recombinant murine IL-4 (Cat. no. 214-14, Peprotech) and 10ng/ml of recombinant murine IL-13 (Cat. no. 210-13, Peprotech) for 24h. For MSC/macrophage assays, Balb/c or C57BL/6 MSCs were added to wells of macrophages at a ratio of 1:5 MSCs/macrophages and co-cultured for 72h. Following co-culture, macrophages were trypsinized, washed in PBS (x4) and cell pellets were stored at -20°C (-80°C if long-term storage was needed) until required (see Supplemental Figure 4A for schematic).

**Generation of single cell suspensions from lymph nodes and spleens and flow cytometry**

Cervical draining lymph nodes (at least 1 large and up to 2 smaller) were identified in the neck region of transplanted mice and removed gently using a forceps and scissors. Briefly, organs were gently mashed through 40μM cell strainers (Thermo-Fisher Scientific) in 6cm petri dishes (Sarstedt, Wexford, Ireland) containing 5ml DPBS. Cell suspensions were then centrifuged at 800 x g for 5 mins. Lymphocytes were washed in DPBS and counted. Splenocytes were re-suspended in ACK lysis buffer (distilled water, 0.15M NH4CL, 10mM KHCO3, Sodium EDTA 0.1mM) and incubated for 5 mins on ice. Reactions were stopped by adding complete medium consisting of RPMI 1640 (Thermo-Fisher Scientific) supplemented with 10% heat-inactivated FBS, 1% sodium pyruvate (1 mmol/l), 1% non-essential amino acids (0.1 mmol/l), 1% L-glutamine (2mmol/l), 1% penicillin (100U/ml)/streptomycin (100μg/ml) and 0.01% β-mercaptoethanol (55μmol/l) (all Sigma-Aldrich). Cells were centrifuged at 800 x g for 5 mins, washed, re-suspended in DPBS and counted.

For flow cytometric analysis, 1x10^5^ cells/sample were stained with the following anti-mouse antibodies diluted in FACS buffer (DPBS supplemented with 1% FBS and 0.05% sodium azide): CD4, CD25, CD69, Foxp3, CD11b, CD11c, MHC II, CD80 and CD206 (all Biolegend) (see Table S1 for antibody catalogue numbers) and with the cell viability dye SYTOX™ AADvanced (Thermo-Fisher Scientific). Unbound antibody was removed by washing twice with FACS buffer. Cells were resuspended in FACS buffer and analysed using a FACS Canto flow cytometer (BD Biosciences, Oxford, UK). For Foxp3 detection, cells were fixed and permeabilised after staining for surface markers using a commercial kit (eBioscience, San Diego, CA) based on the manufacturer’s guidelines. Samples were analysed using a BD FACSCanto II Flow Cytometer (BD Biosciences, California, USA). Flow cytometry data was analysed using FlowJo analysis software version 10 (Tree Star Inc., OR, USA).

**RNA isolation and RT-PCR**

We performed real time (RT)-PCR analysis on dLN cells from PBS treated control and allogeneic MSC treated corneal allograft recipient mice taken at post-operative day (POD) 2. RT-PCR analysis was also performed on RAW264.7 macrophages alone or following co-culture with Balb/c or C57BL/6 MSCs. RNA was extracted from cell pellets using a Bioline Isolate II RNA mini kit according to the manufacturer’s instructions and quantified by NanoDrop. cDNA was synthesised from 1µg RNA using RevertAidTM H-Minus Reverse Transcriptase (Thermo-Fisher Scientific). mRNA expression analysis was performed using TaqMan Gene Expression Assays (Thermo-Fisher Scientific) for Gapdh (Mm99999915_g1), Arg1 (Mm00475988_m1) or Tgfb1 (Mm01178820_m1) and an Integrated DNA Technologies (Leuven, Belgium) PrimeTime Mini qPCR Assay for β2-microglobulin (Mm.PT.58.10497647). Quantitative RT- PCR was performed according to the standard program on the StepOnePlus RT-PCR system (Thermo-Fisher Scientific). mRNA expression levels were normalised to either the housekeeping gene Gapdh or β2-microglobulin and expressed relative to the M0 untreated macrophage group or PBS treated control group, respectively, depending on whether the cells were derived from the dLNs of transplanted mice or *in vitro* cultured RAW264.7 macrophages.

**Statistical analysis**

Statistical analysis was performed using GraphPad Prism Version 8 (La Jolla, USA). Comparisons between two groups were analysed by either non-parametric Mann–Whitney t-test or parametric t-test with Welch’s correction depending on whether the data was normally distributed or not. One-way ANOVA was used to analyse data containing three or more groups, followed by Tukey’s multiple comparison post-test. Data are presented as mean ± SD. Kaplan-Meier survival curves with log-rank (Mantel Cox) test were used for analysis of allograft survival. Differences were considered significant if p ≤ 0.05.

**Figure Legends**

**Supplemental Figure 1. Surface profile characterisation of C57BL/6 MSCs**

Flow cytometry analysis was carried out to confirm MSC surface characterisation for established markers. Shown are representative histograms for cell surface expression of positive (CD44, CD29, CD73, CD105, MHC I, PDGFR-α, PDGFR-β) and negative (F4/80, CD45.2, MHC II, EpCAM) MSC antigens. See Table S1 for antibody information.

**Supplemental Figure 2. Osteogenic and adipogenic differentiation of C57BL/6 MSCs**

C57BL/6 MSCs were cultured in osteogenic or adipogenic induction medium to confirm multilineage potential. (A) Brightfield microscopy images of Alizarin Red S stained osteocytes in control and differentiated MSCs. (B) Calcium content quantification of control MSC compared to MSC cultured in osteogenic medium. (C) Brightfield microscopy images of lipids and triglycerides stained by Oil Red O in MSCs cultured in control or adipogenic medium. (D) Quantification of Oil Red O absorbance in control and adipogenic differentiated MSCs. Error bars: Mean ± SEM. ****p<0.0001. Unpaired, two tailed student’s t test. (n=3). Scale bar in (A) and (C) = 200um.

**Supplemental Figure 3. Dual administration of low-dose allogeneic MSCs does not significantly alter the frequency of mononuclear phagocytes or activated dendritic cells in the spleen**

Spleens were harvested at D+2 from corneal allograft recipient mice receiving either two injections of PBS or low-dose allogeneic MSCs (D-1 and D+1). (A) Proportion of MHCII+ DCs expressed as a percentage of the parent (CD11c+) population. (B) Proportion of CD80+ DCs expressed as a percentage of the parent (CD11c+) population. (C) Proportion of MHCII+ MPh expressed as a percentage of the parent (CD11b+) population. (D) Proportion of CD80+ MPh expressed as a percentage of the parent (CD11b+) population. (E) Proportion of CD206+ MPh expressed as a percentage of the parent (CD11b+) population. Error bars: mean ± SD (each individual dot represents a separate animal, n=3-6). D’Agostino & Pearson omnibus normality test and Shapiro-Wilk normality test used to determine distribution of data. ROUT testing was used to identify outliers. Non-parametric unpaired two-tailed students t tests used for data that was not normally distributed.

**Supplemental Figure 4. C57BL/6 MSCs polarize M0 and skew M1-like macrophages towards an-anti-inflammatory, M2-like phenotype after co-culture**

Non-polarized, M1-polarized (IFN-γ + LPS) or M2-polarized (IL-4 + IL-13) RAW264.7 macrophages (Balb/c-derived) were co-cultured with either syngeneic (Balb/c) or allogeneic (C57BL/6) MSCs for 72 h. Macrophages were collected and pelleted, followed by RNA isolation and cDNA synthesis according to the methods detailed in the relevant section of the Materials and Methods. Arg-1 mRNA expression was assayed as a marker of anti-inflammatroy, M2-like macrophage phenotype. (A) Schematic overview of the process. (B) Arg-1 mRNA fold expression by M0 non-polarized RAW264.7 macrophages alone or after co-culture with Balb/c or C57BL/6 MSCs. (C) Arg-1 mRNA fold expression by M1-polarized RAW264.7 macrophages alone or after co-culture with Balb/c or C57BL/6 MSCs. (D) Arg-1 mRNA fold expression by M2-polarized RAW264.7 macrophages alone or after co-culture with Balb/c or C57BL/6 MSCs. (E) Arg-1 mRNA fold expression by either Balb/c or C57BL/6 MSCs alone. Error bars show mean ± SD. n=3. One-way ANOVA followed by Tukey’s multiple comparison post-test, **p<0.01.

**Supplemental Table**

| **Item** | **Fluorochrome** | **Supplier** | **Cat. No.** |
| --- | --- | --- | --- |
| CD4 | APC | Biolegend, California, USA | 100412 |
| CD25 | BV421 | Biolegend, California, USA | 102034 |
| CD69 | PE | Biolegend, California, USA | 104508 |
| Foxp3 | AF488 | Biolegend, California, USA | 126406 |
| CD11b | FITC | Biolegend, California, USA | 101205 |
| CD11c | FITC | Biolegend, California, USA | 117306 |
| Biotin MHCII (I-A^d^) | APC-Strep | Biolegend, California, USA | 115003 |
| CD206 | PE | Biolegend, California, USA | 141706 |
| CD80 | BV421 | Biolegend, California, USA | 104725 |
| CD44 | PE-Cy7 | Biolegend, California, USA | 103030 |
| CD29 | PE | Biolegend, California, USA | 102208 |
| CD73 | APC | Biolegend, California, USA | 127210 |
| CD105 | APC | Biolegend, California, USA | 120414 |
| CD45.2 | APC | Biolegend, California, USA | 109814 |
| F4/80 | FITC | Biolegend, California, USA | 123108 |
| MHCI (H-2k^b^) | PE-Cy7 | Biolegend, California, USA | 116520 |
| CD140a (PDGFR-α) | APC | Biolegend, California, USA | 135908 |
| CD140b (PDGFR-β) | PE | Biolegend, California, USA | 136006 |
| CD326 (EpCAM) | FITC | Biolegend, California, USA | 118208 |
| Biotin MHCII (I-A^b^) | APC-Strep | Biolegend, California, USA | 116403 |
| Rat IgG1, κ Isotype Ctrl | APC | Biolegend, California, USA | 400411 |
| Rat IgG2a, κ Isotype Ctrl | APC | Biolegend, California, USA | 400511 |
| Mouse IgG2a, κ Isotype Ctrl | PE/Cy7 | Biolegend, California, USA | 400253 |
| Rat IgG2b, κ Isotype Ctrl | PE/Cy7 | Biolegend, California, USA | 400617 |
| Biotin Mouse IgG2a, κ Isotype Ctrl | APC-Strep | Biolegend, California, USA | 400203 |
| Mouse IgG2a, κ Isotype Ctrl | APC | Biolegend, California, USA | 400222 |
| Armenian Hamster IgG Isotype Ctrl | PE | Biolegend, California, USA | 400908 |
| Rat IgG2a, κ Isotype Ctrl | FITC | Biolegend, California, USA | 400506 |
| Rat IgG2a, κ Isotype Ctrl | APC | Biolegend, California, USA | 400512 |
| Rat IgG2a, κ Isotype Ctrl | PE | Biolegend, California, USA | 400508 |
| Streptavidin | APC | Biolegend, California, USA | 405207 |
| SYTOX™ AADvanced™ | PerCp | Thermo-Fisher Scientific, Dublin, Ireland | S10274 |

**Table S1. Antibodies used for flow cytometry.**
