# Supplementary figures and images for: Subconjunctival administration of low-dose murine allogeneic mesenchymal stromal cells promotes corneal allograft survival in mice
Source: Stem Cell Res Ther. 2021 Apr 6;12:227. doi: 10.1186/s13287-021-02293-x (PMC8025388; doi:10.1186/s13287-021-02293-x)

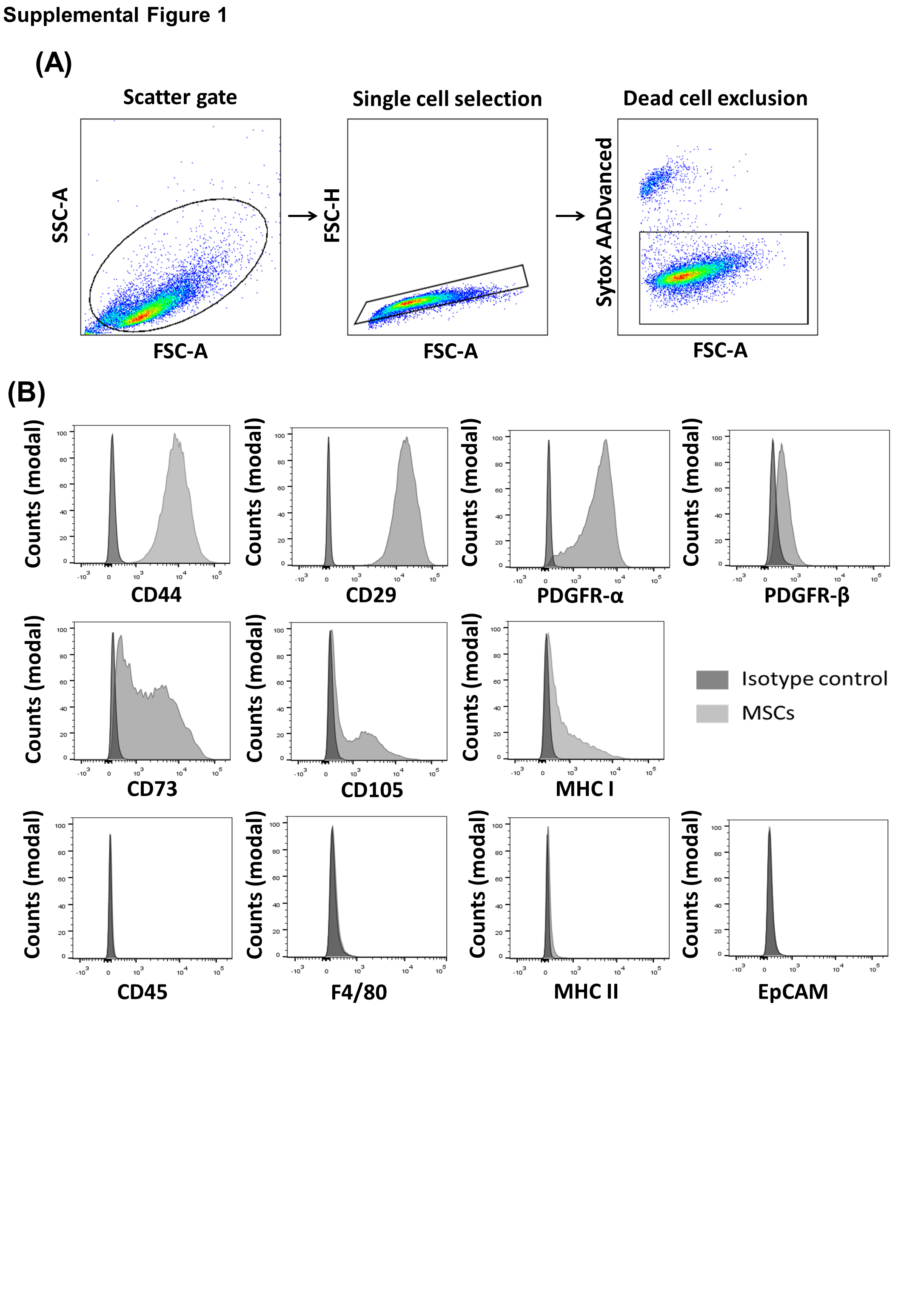

Supplement: Supplementary file 2 — Additional file 2: Supplemental Figure 1. Surface profile characterization of C57BL/6 MSCs. [file 13287_2021_2293_MOESM2_ESM.tif]

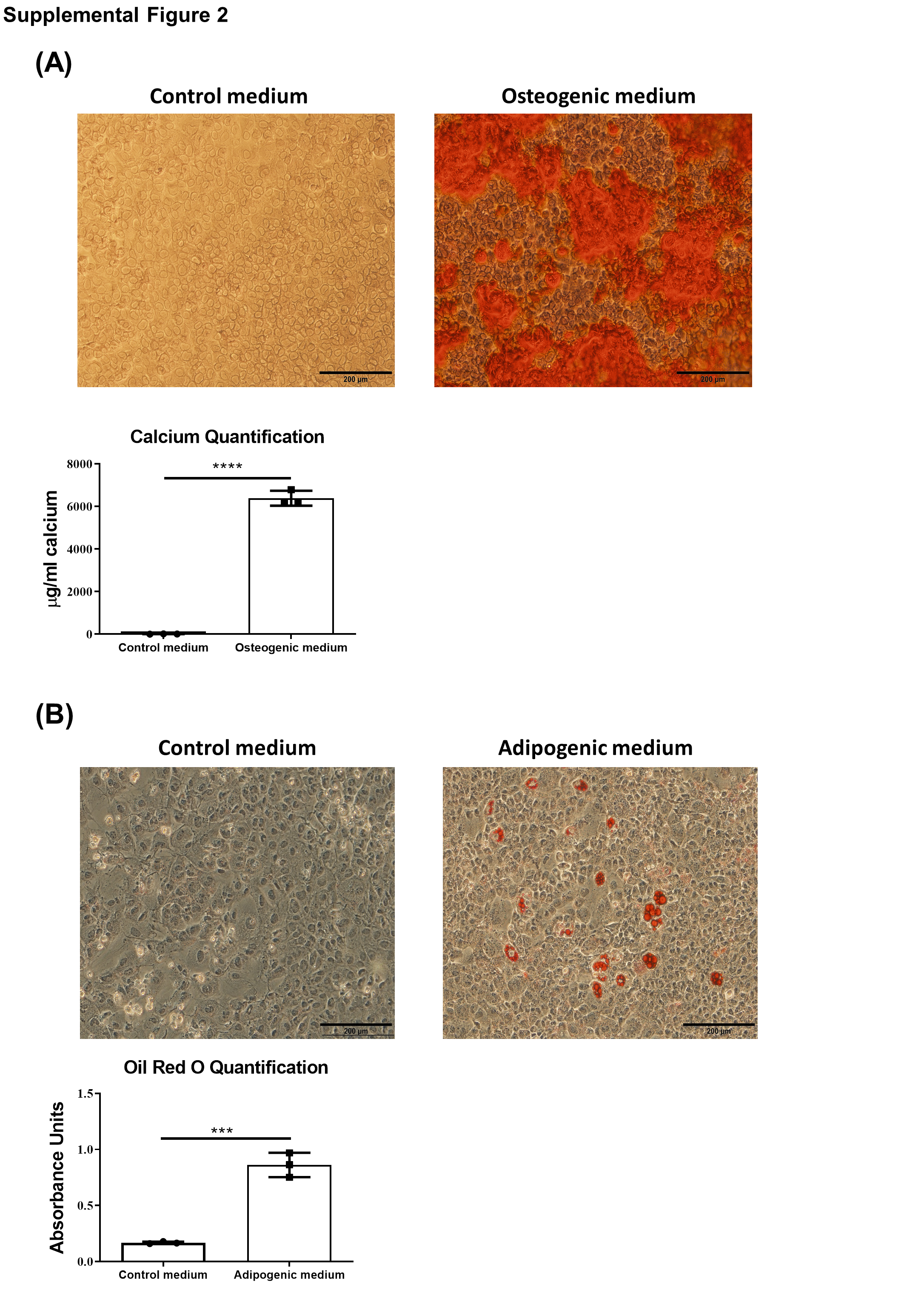

Supplement: Supplementary file 3 — Additional file 3: Supplemental Figure 2. Osteogenic and adipogenic differentiation of C57BL/6 MSCs. [file 13287_2021_2293_MOESM3_ESM.tif]

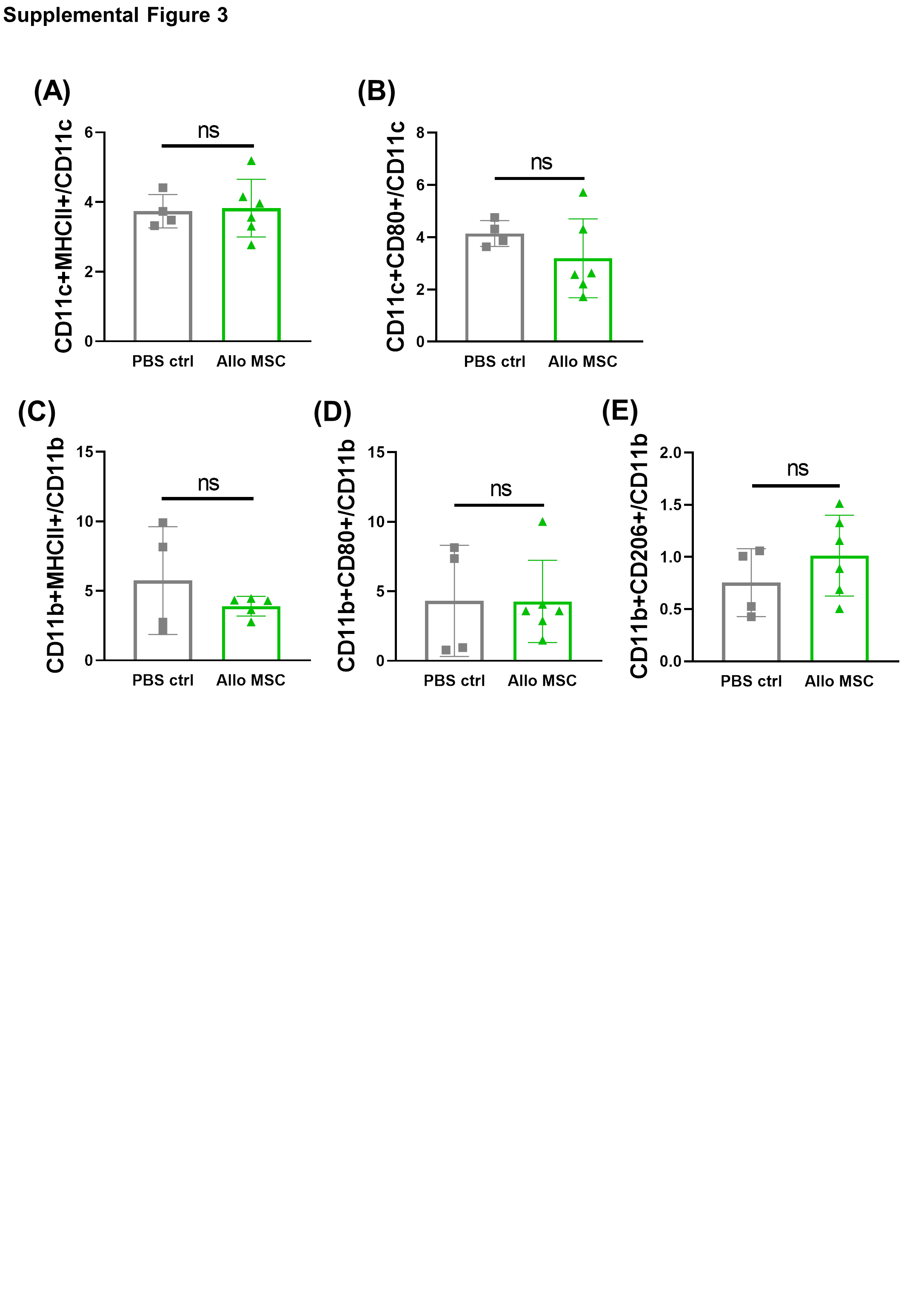

Supplement: Supplementary file 4 — Additional file 4: Supplemental Figure 3. Dual administration of low-dose allogeneic MSCs does not significantly alter the frequency of mononuclear phagocytes or activated dendritic cells in the spleen. [file 13287_2021_2293_MOESM4_ESM.tif]

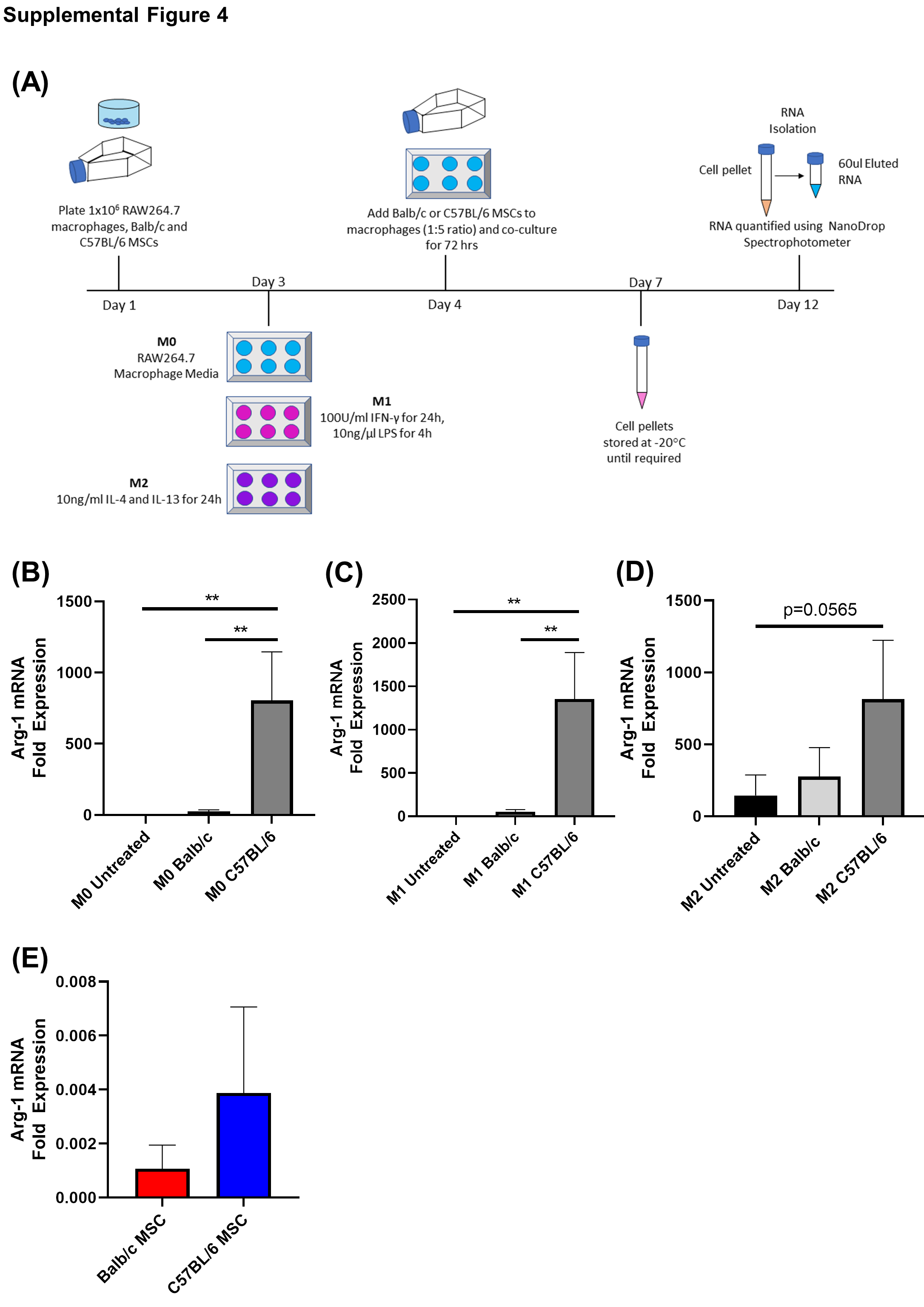

Supplement: Supplementary file 5 — Additional file 5: Supplemental Figure 4. C57BL/6 MSCs polarize M0 and skew M1-like macrophages towards an-anti-inflammatory, M2-like phenotype after co-culture. [file 13287_2021_2293_MOESM5_ESM.tif]
